# Coal and Gangue Detection Networks with Compact and High-Performance Design

**DOI:** 10.3390/s24227318

**Published:** 2024-11-16

**Authors:** Xiangyu Cao, Huajie Liu, Yang Liu, Junheng Li, Ke Xu

**Affiliations:** 1 Collaborative Innovation Center of Steel Technology, University of Science and Technology Beijing, Beijing 100083, China; clovey_cxy@163.com (X.C.); lhjk2s@163.com (H.L.); leosea88@gmail.com (Y.L.); lijunheng0906@163.com (J.L.); 2 Hebei Puyang Iron & Steel Co., Ltd., East of Yangyi Town, Wu’an City 056305, China

**Keywords:** coal–gangue detection, object distribution density measurement (ODDM), relative resolution object scale measurement (RROSM), label rewriting problem, compact neural network

## Abstract

The efficient separation of coal and gangue remains a critical challenge in modern coal mining, directly impacting energy efficiency, environmental protection, and sustainable development. Current machine vision-based sorting methods face significant challenges in dense scenes, where label rewriting problems severely affect model performance, particularly when coal and gangue are closely distributed in conveyor belt images. This paper introduces CGDet (Coal and Gangue Detection), a novel compact convolutional neural network that addresses these challenges through two key innovations. First, we proposed an Object Distribution Density Measurement (ODDM) method to quantitatively analyze the distribution density of coal and gangue, enabling optimal selection of input and feature map resolutions to mitigate label rewriting issues. Second, we developed a Relative Resolution Object Scale Measurement (RROSM) method to assess object scales, guiding the design of a streamlined feature fusion structure that eliminates redundant components while maintaining detection accuracy. Experimental results demonstrate the effectiveness of our approach; CGDet achieved superior performance with AP50 and AR50 scores of 96.7% and 99.2% respectively, while reducing model parameters by 46.76%, computational cost by 47.94%, and inference time by 31.50% compared to traditional models. These improvements make CGDet particularly suitable for real-time coal and gangue sorting in underground mining environments, where computational resources are limited but high accuracy is essential. Our work provides a new perspective on designing compact yet high-performance object detection networks for dense scene applications.

## 1. Introduction

Coal, as a cornerstone of global economic development, serves dual roles as an essential energy source and critical chemical raw material [1]. The imperative to address environmental concerns while maintaining coal’s economic utility has led to the emergence of green and intelligent mining technologies [2]. These technologies represent a significant advancement in sustainable mining practices, integrating underground gangue separation with sophisticated backfilling techniques to minimize environmental impact and prevent mining-induced geological hazards [3]. Such integration not only reduces surface pollution from coal preparation facilities but also effectively mitigates the risk of ground subsidence, marking a substantial advancement in sustainable mining practices. A critical challenge in implementing green mining technologies lies in the spatial constraints of underground operations, which preclude the use of conventional surface processing equipment [4,5]. The dimensional limitations and operational complexities of traditional surface equipment pose significant barriers to underground deployment, necessitating innovative solutions for in situ coal processing. While intelligent sorting robots offer a promising solution due to their compact design [6], their effectiveness fundamentally depends on accurate machine vision systems for real-time coal and gangue discrimination [7]. The development of efficient and accurate machine vision methods is crucial for enabling these robots to perform swift and precise gangue removal during raw coal transportation, thereby facilitating the transition toward environmentally sustainable coal production practices.

Recent developments in convolutional neural networks have demonstrated remarkable potential in object detection tasks [8,9,10], particularly in coal–gangue discrimination applications [11]. Bounding boxes are used by object detection algorithms based on convolutional neural networks to identify the category and location of objects in images [12,13,14]. The evolution of deep learning architectures has revolutionized machine vision capabilities, enabling unprecedented accuracy in object detection and classification tasks. However, the application of these technologies in underground mining environments presents unique challenges that current solutions have yet to adequately address. Existing approaches primarily fall into two categories: two-stage detectors and single-stage detectors. Two-stage detectors, exemplified by Faster R-CNN [15], CG-RPN [16], and FCCN [17], achieve impressive accuracy through sophisticated proposal generation mechanisms but suffer from substantial computational overhead that impedes real-time processing capabilities [7]. These algorithms, while effective in controlled environments, face significant challenges in meeting the speed requirements of online sorting applications. Conversely, single-stage detectors like YOLO [18] offer enhanced processing efficiency but frequently compromise on detection accuracy [19]. Various optimization attempts, including cascaded architectures combining YOLOV3 with support vector machines [20] and implementations of deformable convolutions [21], have been proposed to address these limitations. However, these approaches have not fully resolved the fundamental speed-accuracy trade-off, particularly in dense scene detection scenarios.

The subsequent evolution of YOLOv3 variants, incorporating more sophisticated feature extraction networks and optimized training methodologies, has yielded significant improvements in coal and gangue perception accuracy [22,23,24,25]. These advanced models demonstrate enhanced detection capabilities but are characterized by large parameter spaces and substantial computational requirements, presenting significant challenges for deployment on edge devices with limited resources. This limitation has catalyzed research interest in lightweight object detection models [26,27]. Current model lightweighting approaches can be broadly categorized into two types [28]. The first is network architecture design, which includes manual design [29,30,31,32] and AutoML design [33]. The second is model compression, achieved through techniques such as network pruning [34], low-rank decomposition [35,36], low-bit quantization [37], and knowledge distillation [38]. Contemporary approaches to model compaction have primarily focused on network substitution strategies, such as replacing heavyweight networks with lighter alternatives. Common approaches include substituting DarkNet53 with ResNet18 [39] or MobileNetV3 [20] in YOLOv3 implementations, or replacing VGG16 with GhostNet [40] or MobileNetV1 [41] in SSD (Single Shot MultiBox Detector) architectures [42]. SSD is a single-stage object detection algorithm optimized based on the VGG16 architecture. It leverages feature maps at multiple levels for multi-scale detection, employing convolutional operations and predefined anchor boxes. While these modifications effectively reduce model parameters and computational demands, they often result in compromised detection performance, particularly in challenging dense-scene scenarios. To improve the performance of lightweight models, attention mechanisms are usually added, but adding attention mechanisms does not help to solve the label rewriting problem. Using a larger input image or feature map resolution for detection can alleviate the label rewriting problem, but blindly increasing the resolution of the input image and feature map will increase the computational complexity and seriously weaken the inference speed of the lightweight model [40,43]. In compact convolutional neural networks designed for recognizing coal and gangue, many high-performing models have emerged [44,45,46,47,48]. However, these models often overlook the dense distribution of coal and gangue, as well as the relatively small proportion of pixels occupied by coal and gangue in the images. Therefore, when compacting convolutional neural networks, how to continuously subtract so that they can maintain high performance and speed in dense scenarios while avoiding label rewriting is an important research question.

Recent advances in multi-scale feature learning and multi-view analysis have provided valuable insights for dense object detection. Wang et al. proposed a progressive learning strategy with multi-scale attention network, demonstrating the importance of scale-adaptive feature extraction [49]. Similarly, Wang et al. introduced a bi-consistency guided approach for incomplete multi-view clustering, highlighting the significance of consistent feature representation [50]. Furthermore, Wang et al. developed a graph-collaborated auto-encoder framework for multi-view clustering, offering novel perspectives on feature fusion [51]. Building upon these works, our CGDet advances the field by introducing ODDM and RROSM methods specifically designed for dense coal–gangue detection, while maintaining computational efficiency through optimized feature fusion strategies.

The current state of research faces three critical challenges that previous studies have failed to adequately address: (1) Performance Degradation in Dense Scenes: existing lightweight models struggle to maintain detection accuracy when confronted with densely distributed objects, a common scenario in coal–gangue sorting applications. (2) Computational Overhead: the integration of attention mechanisms and other performance-enhancing features often introduces significant computational burden, contradicting the primary goal of achieving a lightweight model. (3) Label Rewriting Issues: the problem of label rewriting becomes particularly acute in high-density scenes, where multiple objects compete for detection resources within limited spatial regions.

To address these fundamental challenges, this paper introduces CGDet, a novel lightweight convolutional neural network specifically designed for dense coal–gangue detection. Our approach introduces two innovative methodologies: (1) Object Distribution Density Measurement (ODDM): A systematic approach for analyzing and optimizing object detection in dense distributions. This methodology enables precise calibration of input image and feature map resolutions while maintaining high performance and computational efficiency. (2) Relative Resolution Object Scale Measurement (RROSM): A novel technique for characterizing object scale variations and optimizing feature fusion structures. This approach facilitates the development of efficient multi-scale detection strategies while minimizing computational requirements.

The primary objective of this research was to develop a high-performance, computationally efficient object detection system capable of accurate coal–gangue discrimination in dense underground mining environments. Specifically, we aimed to: (1) design a lightweight model architecture that maintains high detection accuracy in dense scenes while minimizing computational requirements. (2) Develop novel methodologies for optimizing input resolution and feature fusion based on object distribution characteristics. (3) Demonstrate the effectiveness of our approach through comprehensive experimental validation.

The primary innovations and contributions of this research are synthesized into three interconnected aspects: (1) The Object Distribution Density Measurement (ODDM) methodology is proposed, enabling optimal resolution selection, circumventing label rewriting issues, and providing a systematic analytical framework for object distribution patterns, with both theoretical foundations and practical guidance established for parameter optimization in dense detection scenarios. (2) The Relative Resolution Object Scale Measurement (RROSM) technique is introduced, facilitating the optimization of feature fusion design through precise quantification of object scale variations, with model complexity significantly reduced and detection accuracy maintained. (3) Based on these innovations, the CGDet architecture is constructed, integrating the advantages of ODDM and RROSM within a unified framework.

Optimal performance is achieved in dense coal–gangue detection tasks, while computational efficiency is preserved for edge device deployment, offering a viable solution for practical engineering applications. Together, these innovations form a cohesive technical system, establishing a new paradigm for lightweight object detection in dense environments.

## 2. Materials and Methods

### 2.1. YOLOX Object Detector

**Figure 1:**
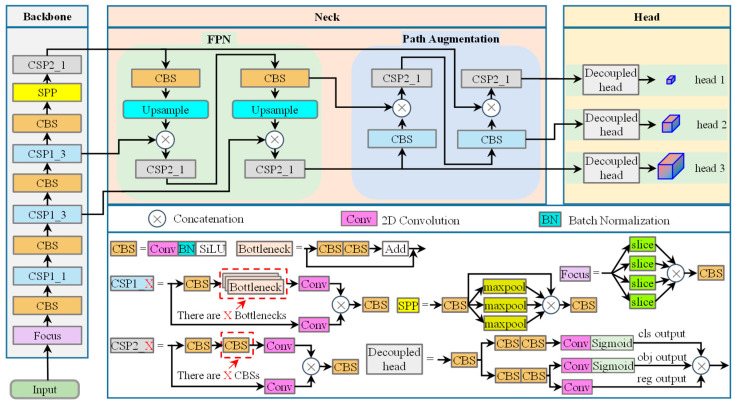
Structure of the YOLOX-s model.

YOLOX [52] is one of the state-of-the-art single-stage object detectors known for its fast detection speed and high accuracy. It has been widely utilized in object detection tasks and it is advantageous for real-time and high-precision perception of coal and gangue in dense scenes. The YOLOX detector still follows the YOLO detection paradigm, which involves gridding the image, and if an object’s center is within a grid cell, that grid cell is responsible for detecting the object. The structure of the YOLOX-s model is shown in Figure 1. As shown in Figure 1, the YOLOX-s model encompasses three main parts: the backbone is used to extract features from the image, the neck is used to fuse feature maps at different scales, and the heads use the feature maps generated by the neck for detection. The backbone of the YOLOX-s model primarily consists of the Focus, CBS, CSP1_X, and SPP (Spatial Pyramid Pooling) modules. Through the Focus layer, the image is sliced, resulting in a reduction of its resolution. The CBS module amalgamates 2D convolution, batch normalization, and SiLU activation functions, encompassing an effective combination. The bottleneck layer incorporates CBS modules and assumes the responsibility of extracting features. The CSP1_X module serves as a feature extraction unit, where X denotes the count of bottleneck layers. For instance, CSP1_3 comprises three bottleneck layers, while CSP2_1 contains one CBS module. The PAN [53] (Path Aggregation Network) structure is an extension of the FPN [54] (Feature Pyramid Network) and is integrated into the neck of the YOLOX-s model. Feature fusion is achieved through CSP2_X, with X implying the number of CBS modules utilized. The heads of the YOLOX-s model encompass three decoupled heads, namely head 1, head 2, and head 3. These heads are responsible for generating the detection outputs.

### 2.2. Definition of the Label Rewriting Problem

**Figure 2:**
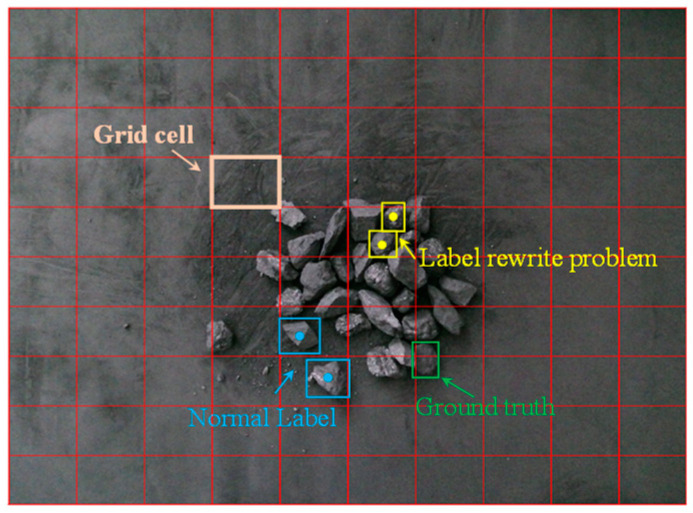
Illustration of CGDet model meshing and label rewriting.

When a large number of objects are densely distributed in an image, the label rewriting problem occurs if the centers of two actual ground truth bounding boxes are located within the same image grid cell. As shown in Figure 2, the yellow color represents labels that have the label rewriting problem, while the blue color represents normal labels. The label rewriting problem can lead to a decrease in the detection performance of the CGDet model. Consider a set B = {b1, …, bt} consisting of the center coordinates of the ground truth bounding boxes in the image, where t represents the total number of ground truth bounding boxes within the image. The label will undergo rewriting when the following condition is fulfilled:

(1) $$\exists (b_{i},b_{j}\in C):b_{i}^{x}\%w-b_{j}^{x}\%w=0,\;b_{i}^{y}\%h-b_{j}^{y}\%h=0$$

where *b_i_* represents the center coordinate of the *i*-th ground truth bounding box, *b_j_* represents the center coordinate of the *j*-th actual ground truth bounding box. $b_{i}^{x}$ and $b_{i}^{y}$ denote the *x* and *y* coordinates of the center of the *i*-th ground truth bounding box. $b_{i}^{x}$ and $b_{i}^{y}$ represent the *x* and *y* coordinates of the center of the *j*-th ground truth bounding box. *w* is the number of columns in the grid, and *h* is the number of rows in the grid.

Label rewriting introduces several negative impacts: (1) detection performance degradation, as some objects may be missed; (2) reduced localization accuracy due to competition among multiple objects within the same grid cell; (3) lower classification accuracy caused by feature interference between adjacent objects; and (4) unstable training performance, as label reassignment affects loss function computation. Therefore, it is essential to mitigate these impacts.

### 2.3. Measure the Distribution Density of Objects in Images

To mitigate the adverse effects of label rewriting on CGDet’s performance, the problem of label rewriting is transformed into a problem of measuring object distribution density. By selecting images with low object distribution density and low resolution for detection, label rewriting can be prevented. Using the ODDM method [55] to calculate the object distribution density in images allows the detector to determine high-performance, low-computation input image resolution, and feature map resolution, thereby improving detector performance while reducing computational load. The calculation formula for ODDM is shown in Equation (2).

(2) $$\beta =\frac{1}{n}\sum_{i=1}^{n}\frac{img_{i}^{g}-img_{i}^{d}}{img_{i}^{g}}$$

where *n* denotes the number of images, $img_{i}^{g}$ denotes the number of objects in the *i*-th image, On the feature map of the *i*-th image, $img_{i}^{d}$ represents the amount of grids which contain objects, *β* represents the density level. A larger *β* value leads to poorer detection performance of the model.

### 2.4. Measure the Scale of an Object in an Image

To optimize the neck structure based on the object scale within the image, the scale of the objects has to be measured. The COCO dataset [56] employs an absolute image resolution to measure object scale, which, unfortunately, leads to inaccurate measurements when dealing with images of varying resolutions. Therefore, RROSM [55] was introduced in this study to accurately capture object scales, which aligns with the scale classification criteria utilized in the COCO dataset. RROSM is calculated as follows.

(3) $$\left\{\begin{array}{c} S=\left[s_{1},s_{2},\cdots ,s_{n}\right]\\ X=\left[\frac{1}{x_{1}},\frac{1}{x_{2}},\cdots ,\frac{1}{x_{n}}\right]\\ G=\left\{g_{ij}\right\}n\times m \end{array}\right.$$

(4) α=X⋅S⋅G

For *n* images, the multiplication of the width and height of the input resolution of each image is computed to obtain the vector *S*. At the original resolution of each image, the reciprocal of the multiplication of width and height produces the vector *X*. The matrix *G* is composed of the areas of the actual bounding boxes for objects in all images, where *i* ∈ {1, 2, …, *n*} and *j* ∈ {1, 2, …, *m*}. The maximum value of object quantity in the images is represented by *m*. Each element *g_ij_* symbolizes the multiplication of the width and height of the actual bounding box of a specific object. If 0 < *a_ij_* <= 32^2^, it is classified as a small object. If 32^2^ < *a_ij_* <= 96^2^, it is classified as a medium object, and if *a_ij_* > 96^2^, it is classified as a large object. When the majority of objects are small, using only the P3 feature map for detection can yield good results.

### 2.5. The Structure of the CGDet Model

Through theoretical analysis, although YOLOX-s exhibits relatively small parameters and computational costs, its structure still presents optimization potential. Based on feature representation theory in deep learning, model performance demonstrates significant correlation with feature map resolution and object distribution density. Consequently, we propose the density theory-based ODDM method to calculate β values, quantitatively evaluating the impact of object distribution on feature extraction. When β values decrease significantly, indicating optimal object distribution density, the corresponding resolution is selected for training and detection, effectively mitigating label rewriting issues and enhancing feature learning quality.

Guided by multi-scale feature representation theory, we employ the RROSM method to analyze object scale distribution characteristics. Experimental evidence indicates that in the absence of large-scale objects, high-level feature maps (such as P5) contribute minimally to feature representation, justifying the removal of corresponding detection heads. When datasets predominantly contain medium and small-scale objects, according to feature pyramid theory, utilizing only the high-resolution P3 feature map suffices for comprehensive feature representation capability. Based on these theoretical analyses, we propose the CGDet model through objective-oriented reconstruction of YOLOX-s.

CGDet maintains the original backbone network structure due to its demonstrated efficacy in feature extraction. Quantitative analysis through RROSM reveals relatively concentrated object scale distribution in our application scenario, negating the necessity for complex feature fusion mechanisms. Therefore, based on information entropy theory, we eliminate the computationally redundant PAN structure, adopting a more concise FPN for feature fusion. ODDM density analysis demonstrates optimal feature representation achievable on the P3 feature map, justifying the retention of only head3 for object detection—a design that ensures detection performance while significantly reducing computational complexity. The backbone network’s CSP1_1, CSP1_3, and CSP2_1 layers constitute a multi-level feature extraction structure, with outputs {C2, C3, C4, C5} and downsampling strides {4, 8, 16, 32} forming progressive feature abstraction levels.

**Figure 3:**
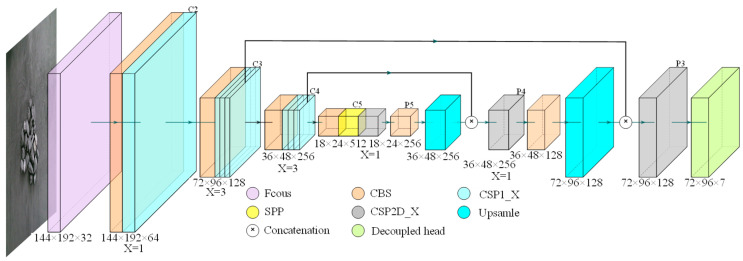
Structure of the CGDet model.

As illustrated in Figure 3, regarding feature fusion, the neck network employs an enhanced FPN structure. Following feature pyramid theory, high-level features (C5) processed through the CBS layer generate semantically rich P5 feature maps. Through upsampling and feature concatenation operations, P5 merges with C4 through CSP2_1 processing to generate P4, achieving effective integration of high and low-level features. Similarly, P4 fuses with C3 to generate P3, establishing a progressive multi-scale feature fusion mechanism. The final feature maps {P3, P4, P5} maintain spatial consistency with {C3, C4, C5}. To further optimize computational efficiency, based on depthwise separable convolution theory, we replace the second CBS in the CSP2_X bottleneck layer with depthwise separable convolution, constructing the FPND structure [32]. This enhancement significantly reduces parameter count and computational complexity while maintaining feature representation capability. The notation X = 1 or X = 3 indicates different levels of feature extraction depth, enabling flexible feature optimization at various levels.

This architectural design, underpinned by solid theoretical foundations, demonstrates the following advantages: (1) optimal resolution selection based on density distribution theory; (2) scale-adaptive feature extraction guided by multi-scale representation theory; (3) efficient feature fusion mechanism supported by information entropy theory; and (4) computational optimization through advanced convolution theories. The integration of these theoretical foundations with practical architectural innovations results in a model that achieves both computational efficiency and detection accuracy in dense object detection scenarios.

## 3. Experiment

### Experimental Environment Settings and Dataset

The experimental hardware resources include an AMD Ryzen 5 3600 CPU and NVIDIA RTX 2060 graphics card. The experiments were conducted on a system running Ubuntu 22.04 LTS with PyTorch 1.9.1, CUDA 10.2, and Python 3.8. In the experiments, the input resolution of the images was set to 512 × 704. The batch size was set to eight, and the initial learning rate was set to 0.003125. The YOLOXWarmCos method was used to update the learning rate during training. The default training duration was set to 300 epochs.

**Figure 4:**
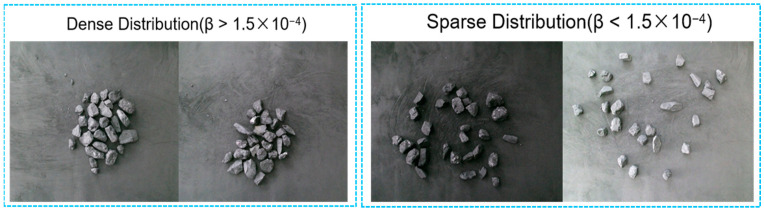
Images of coal and gangue in the dataset.

In the experiment, anthracite and claystone gangue were utilized as the experiment materials. The dataset employed in the experiment is displayed in Figure 4. Image acquisition was carried out using KinectV2 under various lighting conditions, resulting in significant variations in brightness between different images. The contrast between the coal and gangue in the images is relatively low, accompanied by minor differences in surface textures. Moreover, there is a substantial disparity in the distribution of coal and gangue within the images. Whether the objects in an image are densely distributed is determined by calculating the distribution density β using Equation (2) in Section 2.3. A dense distribution is defined as β > 1.5 × 10^−4^, and a sparse distribution as β < 1.5 × 10^−4^. It was observed that 55% of the images in the dataset exhibited dense distributions of coal and gangue, while 45% exhibited sparse distributions. Via random sampling, 400 images from 608 images were taken as the training set, 100 images were taken as the validation set and finally, the remaining 108 images were taken as the test set. All these images were of 1470 × 1080 pixels resolution.

The evaluation of the proposed model encompasses several metrics, including the parameter count, GFLOPs (Giga Floating-point Operations), AP (Average Precision), and AR (Average Recall). AP and AR are computed using the COCO API [52]. Specifically, AP50 and AR50 denote the AP and AR values, respectively, corresponding to an Intersection over Union (IOU) threshold of 0.5. Higher values of AP and AR signify superior model performance.

## 4. Results, Discussion, and Analysis

### 4.1. Ablation Experiments with Different Components

The AP50 and AR50 in this chapter are the results obtained by the model on the test set. The performance comparison of the model on the test set is shown in Table 1.

As shown in Table 1, YOLOX-s had the lowest AP50 in training, with more parameters, computational workload, and inference time. Model A was derived by substituting the original PAN (Path Aggregation Network) structure in YOLOX-s with FPN (Feature Pyramid Network), while Model B was developed through the integration of depthwise separable convolution into the network architecture. Quantitative analysis demonstrates that Model A achieved significant performance improvements over the baseline YOLOX-s architecture: a 28.4% enhancement in mAP_50_ (mean Average Precision at IoU threshold 0.5), while concurrently reducing parameter count by 24.83%, computational complexity by 10.66%, and inference latency by 3.76 ms. Model B achieved a 44.07% reduction in parameters and computation, along with a 4.3 ms faster inference time. Compared to the YOLOX-s baseline, the proposed CGDet demonstrated substantial improvements across multiple performance metrics by achieving a 28.7% increase in mAP_50_, while significantly reducing model parameters and computational complexity by 46.76% and 47.94%, respectively. Furthermore, the model exhibited a 2.9% enhancement in AP50 while decreasing inference latency to 13.61 ms. Using a single detection head on the P3 feature map further reduced parameter, computation, and inference time while maintaining the model’s high-precision detection capability.

### 4.2. Using ODDM to Measure the Distribution Density of Objects in Images

**Figure 5:**
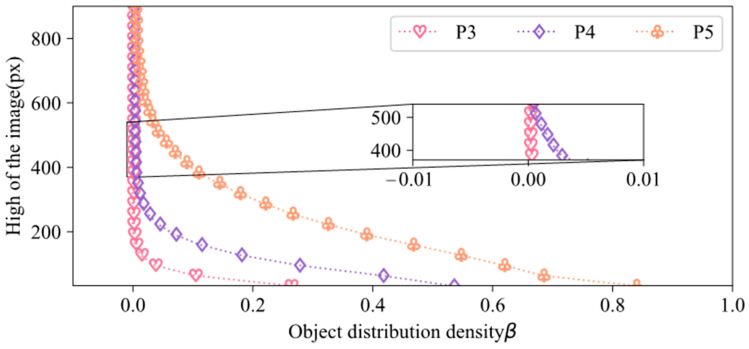
Distribution density of objects in different input resolution images in different resolution feature maps.

To investigate the distribution density of coal and gangue in different resolution feature maps, while maintaining the aspect ratio of the images, the object distribution density in different resolution feature maps was calculated based on Equation (2). The results are shown in Figure 5. The feature maps, denoted as P3, P4, and P5, were obtained by downsampling the input image by 8, 16, and 32 times, respectively. In Figure 5, the height of the image is represented by the vertical axis, which varies from 32 × 224 to 896 × 1088 in resolution. Comparing the feature maps at the same input resolution, the object distribution density was highest in P5 due to its lower resolution, while P3 had the lowest distribution density because of its higher resolution. The object distribution density in P4 fell between that of P3 and P5 since its resolution lay between the two.

As shown in Figure 5, the input image’s resolution rose, and the density of object distribution steadily decreased in the P3, P4, and P5 feature maps. For the P3 feature map, the density gradually decreased beyond an input resolution of 256 × 448. Once the input resolution surpassed 416 × 608, the distribution density of objects decreased at a stable rate. In the zoomed-in section of Figure 5, the object distribution density in P3 had an input resolution of 384 × 576 is 1.64 × 10^−4^, which was an order of magnitude higher than the distribution density of 7.81 × 10^−5^ for an image resolution of 416 × 608. For input resolutions of 416 × 608, 448 × 640, 480 × 672, and 512 × 704, the object distribution density in P3 remained constant. Within the range of 416 × 608 to 512 × 704, the object distribution density was lower in the P3 feature map than in the P4 feature map. The distribution density in the P4 feature map decreased slowly for input image resolutions higher than 640 × 832. P5 consistently had a decreasing density as the input resolution increased from 32 × 224 to 896 × 1088. To counter the impact of densely distributed objects, the CGDet model selects the P3 feature map for detection based on the density results in Figure 5. CGDet uses an image resolution of 512 × 704 for training and detection because the object distribution density is low at this resolution.

### 4.3. Using RROSM to Measure the Scale of Objects in Images

**Figure 6:**
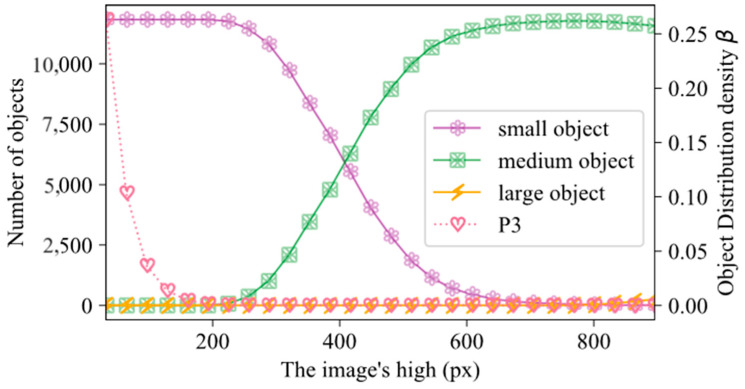
The Scale of objects in the training set.

To enhance the accuracy of measuring the scale of coal and gangue in images, RROSM was employed specifically for the training set. The outcomes obtained from this measurement approach are illustrated in Figure 6, which presents the results derived from the utilization of RROSM. The vertical axis represents the quantities of small, medium, and large objects in the dataset, while the horizontal axis represents the input image resolutions. The image resolution ranges from 32 × 224 to 896 × 1088, and the aspect ratio of the images was maintained during the measurement. Figure 6 depicts the relationship between image resolution and object sizes in the dataset. As resolution increases, small objects decrease while medium objects increase. Small objects transform into medium objects as their resolution on the image grows. Before 416 × 608 resolution, small objects dominate (over 50%), but afterward, medium objects become more prominent. When resolution exceeds 800 × 992, medium objects start transforming into large objects, resulting in a decrease in the number of medium objects and an increase in large objects.

As shown in Figure 6, the y-axis represents the distribution density of objects. Figure 6 depicts the correlation between the input image resolution and the density of object distribution in the P3 feature map. As resolution increases, the distribution density decreases, along with the number of small objects. When the resolution is below 256 × 448, the density is higher, and the dataset is mostly composed of small objects. Model performance is limited by both object distribution density and the presence of small objects at resolutions below 256 × 448.

The distribution density of objects in the P3 feature map decreases as image resolution goes from 256 × 448 to 416 × 608, but small objects still dominate the dataset. From the 416 × 608 to 736 × 928 resolution range, the lowest density of object distribution is in the P3 feature map. At this resolution, the model’s performance is less affected by object distribution density, reducing the impact of small objects on perception. Resolutions above 736 × 928 have almost no small objects and neglectable object distribution density. While higher resolutions improve model performance, training and inference at such resolutions are costly. CGDet uses a 512 × 704 feature map for detection. At this resolution, coal and gangue, which are relatively measured in terms of object scale, are mainly composed of medium and small objects. As deep features are helpful for large object recognition, they are not very helpful for small and medium objects [53]. Therefore, CGDet chooses to use FPN for feature fusion, discarding the path enhancement part in PAN.

To provide additional evidence of the benefits associated with the relative object scale measurement method, Table 2 provides pertinent data regarding the model’s performance and allows for a comparison of the performance between the PAN, FPN, and FPND models.

When the input image resolution is 512 × 704, removing the path enhancement module in PAN, which propagates shallow features to deeper layers, results in no change in the model’s mAP_50_. This suggests that the path enhancement component in PAN is redundant. Eliminating this redundant module improves AP50 and AR50 by 25%, reduces the number of parameters by 25%, decreases computational cost by 8%, and shortens inference time by 19%. FPND employs depthwise separable convolutions within the FPN, achieving reductions in parameter count, computational cost, and inference time at the expense of a slight 0.1% decrease in mAP_50_. Compared to PAN, FPND reduces the number of parameters by 44%, decreases computational cost by 46%, and improves inference speed by 22%. These results further demonstrate that accurately measuring object scale in an image based on relative resolution provides valuable guidance for model architecture design.

### 4.4. Elimination of Redundant Detection Heads via ODDM

To illustrate the negative impact of object distribution density on model perception performance, detection experiments were conducted using feature maps with varying object distribution densities, as shown in the experimental results in Table 3. The P5 feature map, which had the highest object distribution density, impeded the model’s perceptual capability, resulting in AP50, AR50, mAP_50_, and mAR_50_ values all below 90%. In contrast, the P4 feature map, with higher resolution and lower object distribution density, enhanced the model’s perception, increasing AP50 and AR50 by 10.7% and 8.2%, respectively, compared to P5. Furthermore, the model’s mAP_50_ and mAR_50_ improved by 10.7% and 20%, respectively, when using the P4 feature map compared to P5. Increasing the resolution further, the P3 feature map, which had the lowest object distribution density, provided an additional boost to the model’s AP50 and AR50 by 2.9% and 3.3%, respectively, compared to P4. The model’s mAP_50_ and mAR_50_ also increased by 3.5% and 2.8%, respectively, relative to using the P4 feature map. However, using the P3 feature map for detection required additional convolutional layers to fuse the feature maps, leading to increased model parameters and computational complexity. As the resolution of the P5, P4, and P3 feature maps gradually increased, the object distribution density within the feature maps progressively decreased, and the model’s AP50, AR50, mAP_50_, and mAR_50_ steadily improved. Nevertheless, there was a diminishing marginal effect between the improvement in model perception performance and the increase in feature map resolution.

**Figure 7:**
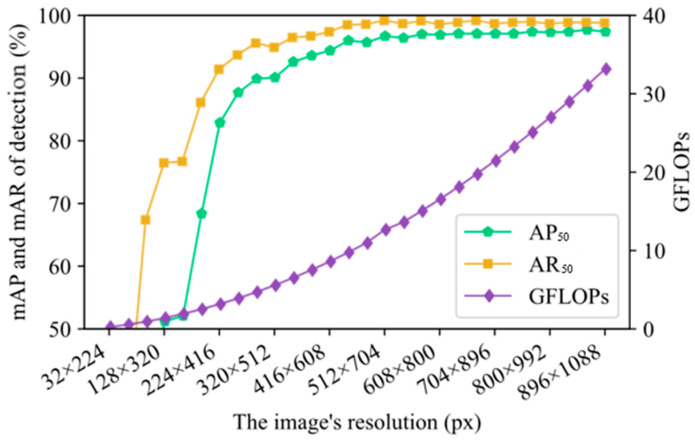
mAP_50_, mAR_50_, and GFLOPs were obtained for images with different input resolutions.

While increasing the input resolution of images or enhancing the resolution of feature maps used for detection can reduce the density of object distribution, the impact of increasing input image resolution and feature map resolution on improving model perceptual performance gradually diminishes. Since CGDet uses the P3 feature map for detection, which has a resolution eight times lower than that of the input image, we conducted experiments to investigate the relationship between input image resolution and model performance. During model training and testing, experiments were conducted with images of different resolutions ranging from 32 × 224 to 896 × 1088, and the results are shown in Figure 7. In Figure 7, as the image resolution increased, the model’s mAP_50_, mAR_50_, and computational load gradually increased. When the image resolution was below 224 × 416, increasing the image resolution significantly improved the model’s mAP_50_ and mAR_50_. When the image resolution ranged from 256 × 448 to 512 × 704, the contribution of increasing image resolution to improving the model’s mAP_50_ and mAR_50_ gradually decreased. Once the image resolution exceeds 512 × 704, the model’s perceptual performance stabilized; further increasing the image resolution hardly improved the model’s performance. Figure 7 reveals a noticeable diminishing return on model performance with increasing image resolution. While higher resolution input images are advantageous in reducing object density and enhancing the resolution of small objects, excessively increasing the input image resolution is counterproductive, leading to a significant increase in redundant computational load. Estimating the density of object distribution in images through methods like object density estimation allows for the determination of high-performance, low-computation image resolutions, thereby reducing the computational burden while maintaining high model performance.

### 4.5. Visualization and Analysis of Results

**Figure 8:**
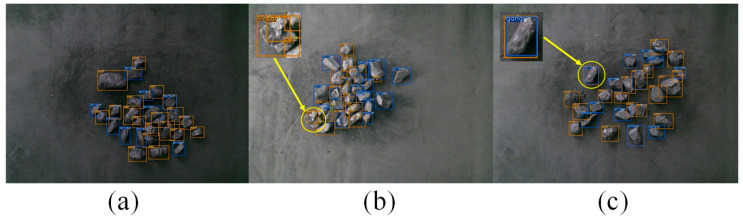
Visualization of CGDet’s detection results on the test set. (**a**) Predicted Bounding Boxes for Gangue (Blue) and Coal (Yellow); (**b**) Redundant Predictions with the Same Class Label (Coal); (**c**) Redundant Predictions with Different Class Labels (Coal and Gangue).

The AP50 and AR50 of CGDet quantitatively represent the performance of the detector. However, they are difficult to observe. Therefore, CGDet was used to detect images in the test set, and the results are visualized in Figure 8.

In Figure 8a, the blue predicted bounding boxes represent gangue, while the yellow predicted bounding boxes represent coal. Most of the predicted boxes cover the corresponding objects in the image, but there are also a few instances where the object is redundantly predicted by two bounding boxes. There are two cases of redundant predictions. In one case, the two boxes with redundant predictions have the same class. As shown in Figure 8b, a piece of coal in the image is simultaneously predicted by two bounding boxes with the class label ‘coal’. In the other case, the two boxes with redundant predictions have different classes. As shown in Figure 8c, the same piece of gangue in the image is predicted as both ‘coal’ and ‘gangue’ by two different bounding boxes. Due to the small area occupied by coal and gangue in the images, this results in a lower amount of discernible surface information for the coal and gangue in images. This makes it more difficult to distinguish them and leads to redundant predictions.

### 4.6. Comparison of the Performance of Different Detectors for Detecting Coal and Gangue

To demonstrate the advantage of CGDet in perceiving coal and gangue in low-resolution dense scenes, comparative experiments were conducted using MMDetection3. The Faster R-CNN, YOLOF, and AutoAssign detectors were utilized for the experiments, all of which employed ResNet50 as the backbone and FPN structure. The batch size in the experiment was eight and the input resolution of the images was set to 512 × 704, using the same dataset and evaluation metrics as CGDet. The results of the comparison experiment are shown in Table 4. Faster R-CNN is an excellent two-stage object detector, and its performance dominance in detecting coal and gangue is not obvious. Although YOLOF also performs detection using a single feature map, its AP50, and AR50 were 0.3% and 2.1% lower than CGDet, respectively. Despite CGDet utilizing only a single feature map for detection, it outperformed YOLOF. AutoAssign employs a dynamic label assignment strategy, but in this experiment, its AP50 and AR50 were 5.7% and 2.4% lower than CGDet, respectively. While YOLOV8n has fewer parameters and computational requirements than CGDet, its performance significantly lagged behind CGDet. YOLOV8s, despite having substantially more parameters and computational demands than CGDet, did not exhibit superior performance either. In this experiment, CGDet demonstrated a clear advantage, achieving AP50 and AR50 values of 96.7% and 99.2%, respectively, in comparison to Faster R-CNN, YOLOF, AutoAssign, and YOLOV8 detectors. Furthermore, CGDet achieved these results with an order of magnitude fewer parameters and significantly reduced computational requirements.

### 4.7. Comparison of Different Coal and Gangue Perception Methods

Many excellent convolutional neural network models have been developed for the classification and localization of coal and gangue in images, but their performances vary. To provide a rough comparison of the performance of these outstanding models, this study selected convolutional neural network models that perceive coal and gangue using color images for comparison. Since these models use different datasets and the source code is not publicly available, the comparative results in Table 5 can only reflect the overall progress in the field.

As shown in Table 5, the proposed CGDet achieved the highest AP50, indicating that CGDet is highly competitive in the perception of coal and gangue. At the same time, CGDet had minimal inference time, indicating its ability to quickly perceive coal and gangue in images. Furthermore, CGDet has fewer parameters and computations, demonstrating its lightweight nature. By comparing with different models listed in the table, it can be seen that many models either suffer from lightweight but poor performance, or good performance but insufficient compacting and longer inference times. CGDet strikes a balance between model compaction and performance, exhibiting outstanding performance and efficiency in perceiving densely distributed coal and gangue in images.

## 5. Conclusions

This paper presents CGDet, a compact convolutional neural network model specifically designed for the perception of coal and gangue in dense scenes. Through extensive experimental validation, the following key scientific and practical findings were established:

Model Performance and Efficiency: CGDet operates with only 4.76 million parameters and 12.26 GFLOPs of computational load, achieving an AP50 of 96.7% and an AR50 of 99.2%. This demonstrates that incorporating object distribution density and scale considerations allows for significant model lightweighting without sacrificing performance, thereby informing the design of efficient deep learning models.

Input Image and Feature Map Selection: The Object Distribution Density Measurement (ODDM) method determined an optimal input image resolution of 512 × 704, utilizing P3 as the feature map for detection. These configurations yielded excellent performance in dense scenarios, underscoring the importance of tailored input and feature map resolutions for object detection to mitigate issues associated with label rewriting.

Structural Optimization and Cost Reduction: By employing the Relative Resolution Object Scale Measurement (RROSM) method to assess object scale and optimizing the model’s neck structure, CGDet achieved a 46.76% reduction in parameters and a 47.94% decrease in computational costs, while slightly enhancing both AP50 and AR50. This indicates that the RROSM method is effective at evaluating object scale, playing a crucial role in structural design and the elimination of redundant parameters.

Practical Recommendations: For the specific task of detecting densely distributed coal and gangue, it is advisable for designers and mechanical engineers to develop customized object detection models, as these may outperform general-purpose detectors. Despite CGDet’s superior performance, it remains susceptible to duplicate detections. Future work should focus on addressing this issue, potentially through the integration of fine-grained classification methods to enhance detection accuracy.

## Figures and Tables

**Table 1:** Ablation experiments with different components.

Model	Improvement Method			Performance					
	FPN	FPND	Head	AP50 (%)	AR50 (%)	mAP_50_ (%)	Parameters (M)	GFLOPs	Inference Time (ms)
YOLOX-s			3	93.8	99.5	69.6	8.94	23.55	19.87
A	√		3	96.5	99.0	98.0	6.72	21.04	16.11
B		√	3	96.7	99.3	97.9	5.00	12.66	15.57
CGDet		√	1	96.7	99.2	98.3	4.76	12.26	13.61

(In Table 1, the “√” symbol indicates that the corresponding module is used or integrated within the model).

**Table 2:** Performance comparison of different neck structures.

Neck	AP50 (%)	AR50 (%)	mAP_50_ (%)	mAR_50_ (%)	Parameters (M)	GFLOPs	Inference Time (ms)
PAN	96.2	98.9	98.0	99.6	8.94	23.55	19.87
FPN	96.5	99.0	98.0	99.6	6.72	21.04	16.11
FPND	96.7	99.3	97.9	99.6	5.00	12.66	15.57

**Table 3:** The impact of feature maps with different levels of density on model performance.

Feature Map	AP50 (%)	AR50 (%)	mAP_50_ (%)	mAR_50_ (%)	Parameters (M)	GFLOPs
P5	83.1	87.7	85.1	77.0	4.35	9.80
P4	93.8	95.9	95.8	97.0	4.68	10.76
P3 (CGDet)	96.7	99.2	98.3	99.8	4.76	12.26

**Table 4:** Performance comparison of different detectors.

Model	AP50 (%)	AR50 (%)	Parameters (M)	GFLOPs
Faster R-CNN	96.4	97.1	41.35	81.66
YOLOF	96.2	98.4	42.36	34.49
AutoAssign	91.0	96.8	36.25	69.54
YOLOV8n	95.6	99.7	3.2	8.7
YOLOV8s	95.6	99.4	11.2	28.6
CGDet	96.7	99.2	4.76	12.26

**Table 5:** Comparison of different coal and gangue perception methods.

Reference	AP50 (%)	Parameters (M)	GFLOPs	Inference Time (ms)
Q. Liu [23]	96.45	-	-	30.67
D. Yang [25]	91.90	6.64	14.30	-
P. Yan [24]	96.00	-	-	19.00
G. Xue [39]	96.27	-	-	21.97
J. Liu [40]	78.50	-	-	28.41
Y. Liu [43]	80.24	5.97	6.83	11.12
B. Zhang [41]	91.33	-	-	40.00
Z. Lv [20]	88.54	-	-	30.20
CGDet	96.70	4.76	12.26	11.96

## Data Availability

The data presented in this study are available on request from the corresponding author.
